# Editorial: Improving visual deficits with perceptual learning

**DOI:** 10.3389/fpsyg.2015.00491

**Published:** 2015-04-21

**Authors:** Gianluca Campana, Marcello Maniglia

**Affiliations:** ^1^Department of General Psychology, University of PadovaPadova, Italy; ^2^Human Inspired Technologies Research Centre – HIT, University of PadovaPadova, Italy; ^3^Centre de Recherche Cerveau et Cognition, Université de Toulouse-UPSToulouse, France; ^4^Centre National de la Recherche ScientifiqueToulouse, France

**Keywords:** myopia, presbyopia, amblyopia, crowding, nystagmus, tRNS, macular degeneration, blindness

The capability of improving performance on visual tasks with practice has been a matter of intense investigation during the last 40 years (Fiorentini and Berardi, 1980; Sagi, 2011). This phenomenon, called perceptual learning, has been proven to occur with virtually any visual skill or stimulus characteristic (Fahle and Poggio, 2002), and to be long-lasting, thus involving neural plasticity at the level of perceptual or even sensory areas (Sagi and Tanne, 1994). Despite this, only recently has perceptual learning started to be considered a useful tool for improving visual functions in clinical populations. This delayed exploitation has possibly been caused by the common finding that learning was highly specific for the trained stimulus attributes (Fiorentini and Berardi, 1980; Ball and Sekuler, 1981; Ahissar and Hochstein, 1996; Schoups et al., 2001; Campana and Casco, 2003; Fahle, 2005), or even for the trained eye or retinal location (Karni and Sagi, 1991), thus resulting impractical for therapeutic purposes. More recently it has become clear that, under specific training conditions, perceptual learning could generalize to other stimuli, tasks and circumstances (McGovern et al., 2012), yielding potential benefits for various types of visual impairments. So far, perceptual learning has been shown to be effective in improving, among other dysfunctions, visual abilities in amblyopia (Levi and Li, 2009; Polat, 2009; Hussain et al., 2012), mild refractive defects (myopia: Tan and Fong, 2008; Camilleri et al., 2014a; presbyopia: Polat et al., 2012), central or peripheral vision loss and cortical blindness (Kasten et al., 1998; Sabel et al., 2005; Huxlin et al., 2009; Chung, 2011; Das et al., 2014), dyslexia (Gori and Facoetti, 2015), and has even been shown to improve the efficacy of other sensory modalities so that they can somehow replace vision (so called sensory substitution) in blind people (Bach-y-Rita and Kercel, 2003; Ortiz et al., 2011).

The goal of this Research Topic is to demonstrate the development of innovative methods, based on perceptual learning, for treating—or at least overcoming some of the deleterious effects of—various visual dysfunctions, from mild deficits such as myopia to complete blindness. New frontier methods should aim at finding the most effective procedures both in terms of perceptual learning and transfer to useful visual functions. This is made possible by combining different techniques aimed at boosting learning or its generalization, such as training with different stimulus features (Xiao et al., 2008; Harris et al., 2012), exploiting multisensory facilitation (Shams and Seitz, 2008) and reinforcement procedures (Seitz and Watanabe, 2009), or combining perceptual learning with non-invasive brain stimulation procedures (Fertonani et al., 2011). Also, in order to achieve the best possible compliance with the patients, shorter and/or more enjoyable trainings (possibly self-administered at home) should be preferred.

For example, while training on either off-the shelf video games (Li et al., 2011; Franceschini et al., 2013), or specifically designed video games involving detection of low contrast stimuli (Deveau et al., 2014a,b) has been shown to improve a range of visual functions (visual acuity, contrast sensitivity, reading skills and even sport performances) both in normally sighted people and people with developmental dyslexia or amblyopia, in the present Research Topic we see that the latter type of video games can also improve visual acuity in participants with refractive defects such presbyopia (Deveau and Seitz, 2014) or reduce crowding (the deleterious effect of nearby elements on target's perception; Levi, 2008) in participants with cortical deficits such as amblyopia (Hussain et al., 2014). While negligible in normal foveal vision, crowding is an important issue also in children with visual impairment accompanied by nystagmus. Reduction of crowding in these children (besides an improvement of near visual acuity, see Huurneman et al., 2013) can be obtained with training on crowded letters, thus producing faster reaction times and an increase of fixation durations (Huurneman and Boonstra, 2014).

Visual functions in participants with mild refractive defects or amblyopia have also been shown to considerably improve with contrast detection trainings (with or without lateral masking) (Tan and Fong, 2008; Levi and Li, 2009; Polat, 2009; Polat et al., 2012; Camilleri et al., 2014a). Here we see how, both in mild myopia and amblyopia, combining a contrast detection training with non-invasive brain stimulation (specifically, transcranial random noise stimulation—tRNS) seems to yield to faster/more effective perceptual learning and transfer to visual acuity and contrast sensitivity (Camilleri et al., 2014b; Campana et al., 2014).

Perceptual learning can also be successfully applied to patients with loss of central vision. Indeed, past research has shown, in sighted participants, how perceptual learning on a contrast detection task with lateral masking was able to reduce crowding at eccentric retinal locations (Maniglia et al., 2011). Here we see how, in patients with macular degeneration, eccentric perceptual learning with a rapid serial visual presentation (RSVP) produces an improvement in reading speed mainly with supra-threshold word durations (above 200 ms) (Coates and Chung, 2014), while a texture discrimination training enhances temporal processing of eccentric stimuli (reflected in shorter stimulus onset asynchrony needed for discrimination), especially when fixation was stable (Plank et al., 2014).

In fact improved temporal processing in areas of residual vision (besides an extension of such areas) in patients with vision loss (hemianopia or quadrantanopia) can be also obtained with the so-called vision restoration therapy, an individualized program providing stimulation at the border of the dysfunctional visual field (Poggel et al., 2014).

Finally, perceptual learning could be useful even for blind people. Blindness often produces an impaired spatial representation in other sensory domains (e.g., Gori et al., 2014a). Here it is shown that blindfolded sighted participants can learn an auditory spatial bisection task, but improvements only occur when a tactile feedback is delivered, indicating that the tactile system can be used to recalibrate the spatial representation in the auditory domain (Gori et al., 2014b). This finding suggests that, also in blind people, auditory spatial representation can be improved via tactile feedback.

To sum up the findings of the present Research Topic, the studies collected here provide the frontline of behavioral and brain stimulation-coupled treatments of a heterogeneous ensemble of visual dysfunctions. Future studies are needed to define the best combination of approaches in order to improve vision with the shortest and most efficacious training, increasing patients' compliance and tailoring the training specifically for each patients' needs.

## Conflict of interest statement

The authors declare that the research was conducted in the absence of any commercial or financial relationships that could be construed as a potential conflict of interest.
